# Serum IgE anti-dsDNA autoantibodies in patients with proliferative lupus nephritis are associated with tubulointerstitial inflammation

**DOI:** 10.1080/0886022X.2023.2273981

**Published:** 2023-12-07

**Authors:** Leran Chen, Mo Yuan, Ying Tan, Minghui Zhao

**Affiliations:** a Renal Division, Department of Medicine, Peking University First Hospital, Beijing, PR China; b Institute of Nephrology, Peking University, Beijing, PR China; c Key Laboratory of Renal Disease, Ministry of Health of China, Beijing, PR China; d Key Laboratory of Chronic Kidney Disease Prevention and Treatment, Ministry of Education of China, Beijing, PR China

**Keywords:** Anti-dsDNA IgE autoantibodies, activity index, interstitial inflammation, proliferative lupus nephritis, systemic lupus erythematosus

## Abstract

Systemic lupus erythematosus (SLE) is an autoimmune disease characterized by the overproduction of multiple autoantibodies. Lupus nephritis (LN), the most common cause of morbidity and mortality, requires early detection. However, only a limited number of serum biomarkers have been associated with the disease activity of LN. Serum IgE anti-dsDNA autoantibodies are prevalent in patients with SLE and may be associated with the pathogenesis of LN. In this study, serum samples from 88 patients with biopsy-proven proliferative LN were collected along with complete clinical and pathological data to investigate the clinical and pathological associations of anti-dsDNA IgE autoantibodies using ELISA. This study found that the prevalence of IgE anti-dsDNA autoantibodies in patients with proliferative LN was 38.6% (34/88). Patients with anti-dsDNA IgE autoantibodies were more prone to acute kidney injury (17/34 vs. 14/54; *p* = .025). Levels of anti-dsDNA IgE autoantibodies were associated with interstitial inflammation (*r* = 0.962, *p* = .017). Therefore, anti-dsDNA IgE autoantibody levels are associated with tubulointerstitial inflammation in patients with proliferative LN.

## 1. Introduction

Systemic lupus erythematosus (SLE) is one of the most common autoimmune diseases in China and is characterized by the production of multiple autoantibodies. Lupus nephritis (LN) is one of the main causes of morbidity and mortality associated with SLE [1] and is the most common cause of secondary glomerulonephritis in China [2]. To date, over 155 autoantibodies have been detected in the sera of patients with SLE, but only a few of them have been associated with LN disease activity [3]. Autoantibodies play a critical role in the initiation and progression of SLE through interactions with self-tissues as well as autoreactive T cells [4]. Since the autoantibodies elevated in SLE patients might be associated with disease initiation or progression, they may represent useful biomarkers for early diagnosis or prognosis prediction [5]. Among them, anti-dsDNA IgG and anti-C1q IgG autoantibodies are associated with disease activity and prognosis of LN [6], and anti-Sm autoantibodies are associated with renal involvement in SLE [7,8]. However, the prevalence of anti-dsDNA IgG autoantibodies or anti-C1q autoantibodies is only approximately 60% in LN patients, and that of anti-Sm autoantibodies is approximately 30% [9]. The limited positive rate restricts the predictive and diagnostic value of autoantibodies in LN patients. Therefore, more autoantibody biomarkers for LN should be identified.

IgE, with a serum level of approximately 150 ng/ml, is the rarest immunoglobulin in human blood. IgE plays a role in the pathogenesis of various autoimmune diseases, including atopic dermatitis, SLE, rheumatoid arthritis, and asthma [10]. A recent study found that IgE autoantibody levels are associated with SLE disease activity. IgE autoantibodies are prevalent in approximately 65% of patients with SLE and 83% of patients with active SLE [9].

Here, we investigated different IgE autoantibodies in the sera of patients with proliferative LN, including anti-dsDNA IgE autoantibodies, anti-C1q autoantibodies, and anti-Sm autoantibodies. The association between IgE autoantibodies and the clinical and histopathological features of LN was also evaluated.

## 2. Methods

### 2.1. Patient and sample collection

Complete clinical and pathological data for 88 patients with renal biopsy-proven proliferative LN diagnosed between 2016 and 2019 at Peking University First Hospital were collected at presentation and during follow-up. All patients met the 1997 revised American College of Rheumatology criteria for SLE [11].

Serum samples were collected from peripheral blood on the day of renal biopsy, and all patients received steroids before renal biopsies. Informed consent for blood sampling and renal biopsy was obtained from each patient. Serum samples from 30 healthy subjects matched for sex and age were also collected as normal controls.

This study was conducted in compliance with the Declaration of Helsinki guidelines. This study was approved by the local ethical committee (Peking University First Hospital, approval number: 2017(1333)).

### 2.2. Clinical evaluation

The clinical and laboratory data for patients with LN were retrospectively analyzed; this patient information included age, sex, hypertension, fever, malar rash, photosensitivity, oral ulcer, alopecia, arthritis, serositis, neurological disorder, anemia, acute kidney injury (AKI), nephrotic syndrome, leukocytopenia, thrombocytopenia, hematuria, pyuria, serum creatinine level, eGFR, and levels of serum C3, albumin, 24 h proteinuria, and anti-dsDNA antibody. We collected the manifestations attributed to SLE activity within 10 days before renal biopsy expect for proteinuria, alopecia, rash, and mucosal ulcers. Acute kidney injury was defined as an increase in serum creatinine by ≥0.3 mg/dl (≥26.5 μmol/l) within 48 h; an increase in serum creatinine to ≥1.5 times baseline, which is known or presumed to have occurred within the prior seven days; or urine volume below 0.5 ml/kg/h for a duration of six hours [12]. The definition of hematuria was >3 red blood cells (RBCs) per high-powered field (HPF), and pyuria was defined as >5 leukocytes per HPF [13]. The eGFR was calculated based on the serum creatinine level following the CKD-EPI eGFR equation [14]. The disease activity of LN patients was assessed using the Systemic Lupus Erythematosus Disease Activity Index (SLEDAI) [15]. The primary endpoint was death, and secondary endpoints were double serum creatinine level, end-stage renal disease (ESRD), or renal transplantation.

### 2.3. Renal histopathology

Renal biopsy specimens were examined using light microscopy, direct immunofluorescence, and electron microscopy. LN was reclassified according to the 2016 International Society of Nephrology/Renal Pathology Society Classification System [16]. Pathological parameters, including the activity index (AI) and chronicity index (CI), were determined by renal pathologists. The AI contains six parameters: karyorrhexis/fibrinoid necrosis, endocapillary hypercellularity, cellular crescents, subendothelial hyaline deposits, leukocyte infiltration, and interstitial inflammation. The CI considers four parameters: glomerular sclerosis, tubular atrophy, fibrous crescents, and interstitial fibrosis. These specific parameters are assessed using distinct categories of 0%, 1–24%, 25–50%, or >50% and then assigned a semiquantitative score ranging from 0 to 3 points, ensuring a comprehensive evaluation [17].

### 2.4. Detection of IgE autoantibodies by enzyme-linked immunosorbent assay

The levels of antibodies against two common nuclear antigens (dsDNA and Sm) were detected using commercial kits (Calbiotech, El Cajon, CA). To detect dsDNA- and Sm-specific IgE autoantibodies, anti-human IgE-HRP (Immunology Consultants Lab Inc., Portland, OR) was used instead of the anti-human IgG-HRP provided with the ELISA kit, and the optical density (OD) was measured at 450 nm.

According to a previous study, human C1q (Sigma-Aldrich, St. Louis, MO) diluted to 5 μg/ml with coating buffer (10 mM sodium carbonate/bicarbonate, pH 9.6) was coated onto the wells of one-half of a polystyrene microtiter plate (Costar, Corning, NY) and kept overnight at 4 °C [18]. The wells in the other half were coated with the coating buffer alone to act as antigen-free wells. The following steps were performed at 37 °C: the plates were blocked with 1% bovine serum albumin (BSA) for 1 h. Diluted plasma samples were added to the wells and incubated for 2 h. Plates were then incubated with alkaline phosphatase-conjugated goat anti-human IgE (Sigma, St Louis, MO) for 1 h. After incubation, p-nitrophenyl phosphate (pNPP, 1 mg/ml; Sigma, St. Louis, MO) was added, and the results were measured using a microplate reader (Bio-Rad 550, Hercules, CA) at 405 nm. The results were recorded as the net OD 405 nm (average value of antigen wells minus average value of antigen-free wells). The binding of the known positive control serum was 100%, and the binding of the tested sera was expressed as a percentage of a known positive sample. The cutoff value was set as the mean ± 2 standard deviation (SD) of the 30 healthy blood donors.

### 2.5. Statistical analysis

Statistical software SPSS version 26.0 (IBM Corp., Chicago, IL) was used for statistical analysis. Nonnormally distributed continuous variables are expressed as the median with the range (25th percentile, 75th percentile), and normally distributed continuous variables are expressed as the mean ± SD. Categorical variables are expressed as numbers (%). For bivariate comparisons, Student’s *t*-test and nonparametric Mann–Whitney’s *U*-test were used for continuous variables, and *χ*^2^ test [2] and Fisher’s exact test were used for categorical variables. Statistical significance was set at *p* < .05.

## 3. Results

### 3.1. General patient data

General patient data are shown in Table 1. Among the 88 patients with LN, 21 were male and 67 were female, with a mean age of 32.2 ± 12.6 years. The mean SLEDAI score was 18.8 ± 6.7, the AI score was 8 (6–11), and the CI score was 2 (0–3). Sixteen out of 88 patients had type III (18.2%) LN, 11 had type III plus V (12.5%), 45 had type IV (51.1%), and 16 had type IV plus V (18.2%).

**Table 1.** General data for patients with proliferative lupus nephritis.

Clinical evaluation		Laboratory assessment	
Fever, no. (%)	16 (18.2)	Leukocytopenia, no. (%)	11 (12.5)
Skin rash, no. (%)	23 (26.1)	Thrombocytopenia, no. (%)	23 (26.1)
Photosensitivity, no. (%)	7 (8.0)	Hematuria, no. (%)	77 (87.5)
Oral ulcer, no. (%)	12 (13.6)	Pyuria, no. (%)	56 (63.6)
Alopecia, no. (%)	23 (26.1)	Anti-nuclear antibody (+), no. (%)	87 (98.9)
Arthralgia, no. (%)	36 (40.9)	Anti-double-stranded DNA antibody (+), no. (%)	75 (85.2)
Serositis, no. (%)	17 (19.3)	Anti-Smith antibody (Sm) (+), no. (%)	23 (26.1)
Neurological disorder, no. (%)	8 (9.1)	Anti-SSA antibody (+), no. (%)	42 (47.7)
Acute kidney injury, no. (%)	31 (35.2)	Anti-SSB antibody (+), no. (%)	9 (10.2)
Anemia, no. (%)	77 (87.5)	Anti-cardiolipin antibody (+), no. (%)	8 (9.1)

### 3.2. Prevalence of IgE autoantibodies in LN patients

**Figure 1.**
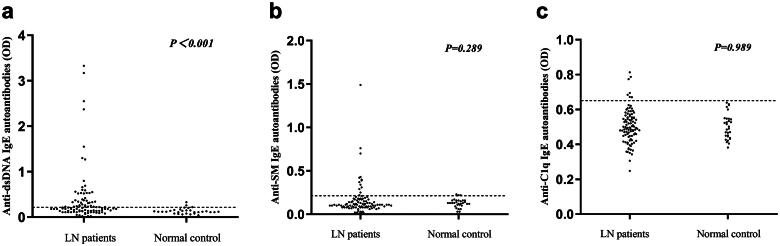
Prevalence of anti-dsDNA IgE, anti-SM IgE, and anti-C1q IgE antibodies in patients with lupus nephritis (LN) and healthy control subjects. The horizontal lines indicate the cutoff value. Levels of serum antibodies against dsDNA IgE, SM IgE and C1q IgE are shown in (a)–(c), respectively.

IgE autoantibody levels were measured in the sera of 88 patients with LN. The cutoff values for anti-dsDNA IgE, anti-Sm IgE, and anti-C1q IgE are illustrated in Figure 1. Anti-dsDNA IgE, anti-Sm IgE, and anti-C1q IgE autoantibodies were detected in 34/88 (38.6%), 12/88 (13.6%), and 6/88 (6.8%) patients, respectively. With a positivity rate of more than one-third, anti-dsDNA IgE antibodies were the most prevalent among the three IgE antibodies, suggesting potential clinical significance.

### 3.3. Clinical associations of anti-dsDNA IgE autoantibodies

Patients with anti-dsDNA IgE autoantibodies had higher SLEDAI scores than those without autoantibodies (18.9 ± 4.3 vs. 18.7 ± 7.9 *p* = .338). Among the 88 patients with proliferative LN, 10 showed neither anti-dsDNA IgG nor IgE autoantibodies, 31 showed both, 44 had only IgG, and three had only IgE autoantibodies. A correlation analysis of anti-dsDNA IgG and IgE antibody levels was conducted in patients with a concomitantly positive IgG and IgE dsDNA, revealing no significant associations between the levels of anti-dsDNA IgG antibodies and IgE antibodies (*r* = 0.261, *p* = .157).

Patients with anti-dsDNA IgE autoantibodies were more prone to AKI (17/34 vs. 14/54; *p* = .025). No significant correlations were observed between anti-dsDNA IgE autoantibodies and other clinical or serological features. Although the role of IgE autoantibodies in the activation of basophils has been confirmed in numerous studies, the levels of serum basophils in patients with positive anti-dsDNA IgE levels were not significantly higher than those in patients without anti-dsDNA IgE autoantibodies (Table 2). Similar analyses were carried out on anti-Sm and anti-C1q IgE autoantibodies and illustrated in Supplementary Table 1. However, due to the low prevalence of anti-Sm and anti-C1q IgE autoantibodies, their clinical significance was marginal.

**Table 2.** Serological and clinical features of patients with lupus nephritis with or without anti-dsDNA IgE autoantibodies.

	Patients with anti-dsDNA IgE autoantibodies (*n* = 34)	Patients without anti-dsDNA IgE autoantibodies (*n* = 54)	*p* Value
Photosensitivity	1 (2.9%)	6 (11.1%)	.241
Alopecia	6 (17.6%)	17 (31.5%)	.213
Arthralgia	11 (32.4%)	25 (46.3%)	.266
Serositis	7 (20.6%)	10 (18.5%)	1.000
Neurological disorder	2 (5.8%)	6 (11.1%)	.477
Acute kidney injury	17 (50.0%)	14 (25.9%)	.025
Anemia	30 (88.2%)	47 (87.0%)	1.000
Leukocytopenia	8 (23.5%)	15 (27.8%)	.804
Thrombocytopenia	8 (23.5%)	15 (27.8%)	.804
Hematuria	30 (88.2%)	47 (87.0%)	1.000
Pyuria	26 (76.4%)	30 (55.6%)	.068
Serum creatinine (µmol/l)	160.86 ± 95.20	144.64 ± 105.57	.468

### 3.4. Pathological associations of anti-dsDNA IgE autoantibodies

The prevalence of anti-dsDNA IgE in patients with type III, type III plus V, type IV, and type IV plus V LN was 37.5% (6/16), 36.4% (4/11), 42.2% (19/45), and 31.3% (5/16), respectively. The AI of patients with or without anti-dsDNA IgE autoantibodies was 9 (6–12.25) vs. 7 (5–10) (*p* = .066), and the CI was 1.5 (0–4) vs. 2 (0–3) (*p* = .804) (Table 3). Semiquantitative scores of both AI and CI in different classes of LN are presented as medians with ranges (25th percentile, 75th percentile) in Table 4, and these parameters were evaluated to investigate the associations between anti-dsDNA antibody levels and active and chronic kidney lesions (Table 3). Levels of anti-dsDNA IgE autoantibodies were associated with interstitial inflammation (*r* = 0.962, *p* = .017).

**Table 3.** Correlations between anti-dsDNA IgE autoantibodies and renal histopathological scores of patients with proliferative lupus nephritis.

	Patients with anti-dsDNA IgE autoantibodies (*n* = 34) median (25th percentile, 75th percentile)	Patients without anti-dsDNA IgE autoantibodies (*n* = 54) median (25th percentile, 75th percentile)	Correlation between AI/CI and anti-dsDNA IgE autoantibodies (*r* value (*p* value))
AI			
AI score	9 (6–12.25)	7 (5–10)	0.197 (0.065)
Karyorrhexis/fibrinoid necrosis	0 (0–2)	0 (0–2)	0.001 (0.822)
Endocapillary hypercellularity	2 (1.75–3)	2 (1–3)	0.008 (1.000)
Cellular crescents	2 (2–4)	2 (0–2)	0.086 (0.099)
Subendothelial hyaline deposits	1 (0–1)	0 (0–1)	0.040 (0.324)
Leukocyte infiltration	1 (0–1.25)	0 (0–1)	0.025 (0.563)
Interstitial inflammation	2 (1–2)	1 (1–2)	0.112 (0.017)
CI			
CI score	1.50 (0–4)	2 (0–3)	0.027 (0.804)
Glomerular sclerosis	0 (0–1)	0 (0–1)	0.013 (0.661)
Tubular atrophy	0.50 (0–1)	1 (0–1)	0.022 (0.627)
Fibrous crescents	0 (0–0)	0 (0–0)	0.023 (0.502)
Interstitial fibrosis	1 (0–1)	1 (0–1)	0.032 (0.449)

**Table 4.** Renal histopathological scores of patients with different classes of lupus nephritis.

Renal pathological types	Type III (*n* = 16)	Type III + V (*n* = 11)	Type IV (*n* = 45)	Type IV + V (*n* = 16)
AI				
AI score	5 (4–6)	6 (3–6)	10 (8–12.5)	10 (7.5–12)
Karyorrhexis/fibrinoid necrosis	0 (0–0)	0 (0–0)	0 (0–2)	0 (0–2)
Endocapillary hypercellularity	1 (1–2)	1 (1–2)	3 (2–3)	2 (2–3)
Cellular crescents	2 (0–2)	2 (0–2)	2 (2–4)	2 (1.25–4)
Subendothelial hyaline deposits	0 (0–0)	0 (0–1)	1 (0–1)	1 (0–1.75)
Leukocyte infiltration	0 (0–0)	0 (0–1)	1 (1–2)	1 (0–2)
Interstitial inflammation	1 (1–2)	2 (1–2)	2 (1–2)	2 (1–2)
CI				
CI score	1.5 (0–4.25)	2 (0–3)	1 (0–3)	3 (0.5–4)
Glomerular sclerosis	0 (0–1)	0 (0–0)	0 (0–0)	0 (0–1)
Tubular atrophy	1 (0–1.75)	1 (0–1)	0 (0–1)	1 (0.25–1)
Fibrous crescents	0 (0–0)	0 (0–0)	0 (0–0)	0 (0–0)
Interstitial fibrosis	0.5 (0–1.75)	0 (0–1)	0 (0–1)	1 (0.25–1.75)

A further investigation into the clinical and serological features of patients with and without interstitial inflammation was conducted (Supplementary Table 2). Patients with interstitial inflammation showed higher serum creatinine (*p* < .001), lower eGFR (*p* = .001), lower hemoglobin levels (*p* = .019), and less anti-cardiolipin antibodies (*p* = .001). Patients with AKI were analyzed separately to highlight the effect of AKI (Supplementary Table 3). No significant associations was found in interstitial inflammation between patients with IgG anti-dsDNA only and patients with both positive IgG and IgE anti-dsDNA antibodies (*Z* = −1.418, *p* = .157).

### 3.5. Associations of anti-dsDNA IgE autoantibodies and composite outcomes

The patients were followed up for a median of 23.5 months (range, 14.0–33.3 months). Using the log-rank test for univariate survival analysis of prognosis in all patients with LN, we found that the positivity of anti-dsDNA IgE autoantibodies was not a risk factor for composite outcomes, including primary and secondary endpoints (hazard ratio (HR), 1.961; 95% confidence interval (CI), 0.487–7.887; *p* = .343).

In a subsequent multivariate Cox hazard analysis of the risk factors, we found that no other covariates were identified as independent risk factors for composite outcomes.

## 4. Discussion

SLE is a common autoimmune disease that affects multiple systems, and renal lesions contribute to the morbidity and mortality of patients with SLE. Research has shown that autoantibodies to dsDNA, especially IgG class autoantibodies, are crucial in the pathogenesis of LN [19,20]. Although the production of anti-dsDNA IgG autoantibodies is frequently detected in patients with LN, they sometimes fail to indicate kidney lesions or disease activity in SLE [21]. Therefore, it is essential to identify novel biomarkers.

Our study confirmed a high prevalence of anti-dsDNA IgE in patients with proliferative LN. Furthermore, the IgE- and IgG-positive groups did not completely overlap, indicating the clinical significance of anti-dsDNA IgE autoantibodies. Existing literature has demonstrated the critical role of IgE in the pathogenesis of autoimmune diseases and the clinical significance of anti-IgE autoantibodies. Previous studies have shown that IgE regulates autoimmune diseases through three major pathways. IgE upregulates the secretion of interferon-α (IFN-α) by activating plasmacytoid dendritic cells (pDCs) [22]. Through the activation of Toll-like receptor 9 (TLR9)-mediated DNA detection in phagosomes, IgE triggers phagocytosis, which in turn activates pDCs and their secretion of IFN-α. Second, IgE can promote the migration of basophils into lymph nodes [23]. Activated basophils in SLE patients upregulate the levels of L-selectin (CD26L), MHC class II, and B-cell activating factor (BAFF) and produce IL-4 and IL-6 [24]. Finally, IgE plays a role in T-cell and B-cell responses. IgE not only triggers the immune responses of CD4+ T cells and activates Th2 cells but also promotes the binding of antigens to MHC I molecules, boosting CD8+ T-cell responses [25]. IgE also participates in B-cell responses by driving B-cell expansion and inducing plasma cell differentiation [22].

Research has shown that anti-IgE autoantibodies may be associated with inflammatory and immediate-type hypersensitivity reactions in autoimmune diseases [26]. IgE autoantibodies are strongly associated with LN [9]. Self-reactive IgE primarily mediates the pathogenesis of LN by promoting inflammatory cell infiltration, especially that of basophils. Basophils promote the differentiation of Th2 cells and upregulate the expression of Th2 signature cytokines and CD40L. Moreover, basophils help maintain the stabilization of plasma cells, enhance the secretion of self-reactive autoantibodies in SLE, and potentially enhance the existing loss of B-cell tolerance [27]. Basophils, IL-4, and IgE work synergistically to mediate the pathological process of LN by upregulating the level of Th2 cells and activating self-reactive B cells. In particular, IgE autoantibodies to dsDNA exacerbate the development and severity of lupus-like disease [28]. Henault et al. proposed that anti-dsDNA IgE autoantibodies affect the onset and activity of LN through mediation of the IFN-α response in pDCs, upregulating the level of pDC IFN-α [22].

We then focused on the pathological correlations of anti-dsDNA IgE autoantibodies and found that patients with anti-dsDNA IgE tended to have a higher AI score than those without, suggesting their possible role in LN. Furthermore, we examined the semiquantitative scores of the histological features of patients and their associations with anti-dsDNA IgE autoantibodies. Positive correlations were confirmed between anti-dsDNA IgE and interstitial inflammation in the activity indices, indicating that IgE might exacerbate the activity of LN by mediating the infiltration of interstitial inflammatory cells. Several studies have shown that IgE facilitates the infiltration of inflammatory cells. Dema et al. found that the absence of IgE leads to a sizable decrease in the infiltration of immune cells in secondary lymphoid organs, which not only significantly affects the levels of dendritic cells, monocytes, neutrophils, and eosinophils in lymphoid organs but also hinders the activation of basophils [9]. Rascio et al. demonstrated that the IgE immune complex promotes the infiltration of basophils and pDCs [29]. The severity of interstitial inflammation was found to be a valid prognostic factor for the deterioration of renal function and positively correlated with the degree of renal lesions [30]. Since the level of autoantibodies to IgE is associated with interstitial inflammation, it might be a potential noninvasive biomarker for interstitial lesions in LN.

Our study reported that anti-dsDNA IgE autoantibodies were positively associated with semiquantitative scores of renal histological features in LN, specifically with the score of interstitial inflammation, which might suggest the essential role of IgE autoantibodies in the infiltration of interstitial inflammatory cells. Although there was no significant difference between the AI or CI scores of patients with and without anti-dsDNA IgE autoantibodies, the mean AI score of the IgE-positive patients was higher. The existing literature has proven that both the composite AI score and individual semiquantitative scores of the activity indices are suggestive of adverse renal outcomes, such as kidney failure [31]. Furthermore, elevation of AI and CI scores has been found to be a prognostic factor for predicting the long-term outcomes of LN [32]. This new finding on the pathological association of anti-dsDNA IgE may shed some light on the pathogenesis of IgE and its autoantibodies in proliferative LN.

However, our study has some limitations. Although positive correlations were found between interstitial inflammation and anti-dsDNA IgE autoantibodies, the specific type of interstitial inflammatory cells affected remains unidentified. Therefore, further research on the role of IgE autoantibodies in inflammatory cell infiltration is needed. Moreover, since anti-dsDNA IgG autoantibodies are currently one of the most common detectors of disease activity, the relationship between anti-dsDNA IgE and IgG autoantibodies needs to be thoroughly investigated. However, owing to the restricted sample size in single center, there was no significant associations between the levels of IgG and IgE dsDNA antibodies in patients with both of them. In addition, there is no difference in the semiquantitative scores of interstitial inflammation between patients with IgG anti-dsDNA only and those with both positive IgG and IgE antibodies. In the future, more samples should be collected and analyzed to clarify the pathological value of IgE autoantibodies.

In conclusion, anti-dsDNA IgE autoantibodies are highly prevalent in patients with proliferative LN and are closely correlated with interstitial inflammation. Anti-dsDNA IgE autoantibodies might serve as a valuable clinical biomarker for disease activity in patients with LN and might also play a role in the pathogenesis of LN and SLE.

## Supplementary Material

Supplemental Material

## Data Availability

There are no datasets associated with this submission.
