# Supplementary material for: Serum IgE anti-dsDNA autoantibodies in patients with proliferative lupus nephritis are associated with tubulointerstitial inflammation
Source: Ren Fail. 2023 Dec 7;45(2):2273981. doi: 10.1080/0886022X.2023.2273981 (PMC11001354; doi:10.1080/0886022X.2023.2273981)
Supplement: Supplemental Material [file IRNF_A_2273981_SM7860.pdf]

Supplementary material

Supplementary table 1: Associations between anti-Sm, anti-C1q IgE aantibodies and clinical and histopathological features of patients with lupus nephritis

	Anti-Sm IgE antibodies (± 12/76), median (range) (*p* value) or prevalence (*p* value)	Anti-C1q IgE antibodies (± 6/82), median (range) (*p* value) or prevalence (*p* value)
Clinical features		
SLEADI	20.33 ± 13.06/18.57 ± 5.16 (0.010)	20.17 ± 4.17/18.71 ± 6.86 (0.910)
Photosensitivity	0 (0.0%)/7 (9.2%) (0.273)	0 (0.0%)/7 (8.5%) (0.456)
Alopecia	4 (33.3%)/19 (25.0%) (0.541)	0 (0.0%)/23 (28.0%) (0.131)
Arthralgia	5 (41.7%)/31 (40.8%) (0.954)	3 (50.0%)/33 (40.2%) (0.639)
Serositis	3 (25.0%)/14 (18.4%) (0.592)	2 (11.8%)/15 (18.3%) (0.368)
Neurological disorder	1 (8.3%)/7 (9.2%) (0.922)	1 (16.7%)/7 (8.5%) (0.504)
Acute kidney injury	3 (25.0%)/28 (36.8%) (0.425)	5 (83.3%)/26 (31.7%) (0.011)
Anemia	10 (83.3%)/67 (88.2%) (0.639)	5 (83.3%)/72 (87.8%) (0.749)
Leukocytopenia	0 (0.0%)/11 (14.5%) (0.159)	0 (0.0%)/11 (13.4%) (0.338)
Thrombocytopenia	2 (16.7%)/21 (27.6%) (0.422)	3 (50.0%)/20 (24.4%) (0.168)
Hematuria	9 (75.0%)/68 (89.5%) (0.159)	6 (100.0%)/71 (86.6%) (0.338)
Pyuria	7 (58.3%)/49 (64.5%) (0.681)	5 (8.9%)/51 (62.2%) (0.299)
Serum creatinine (μmol/l)	136.54 ± 29.52/153.18 ± 11.68 (0.331)	149.68 ± 15.30/151.00 ± 11.56 (0.408)
Histopathological features		
AI		
AI score	7 (6–9.75)/8.5 (6–11) (0.840)	10.5 (7.75–13.5)/8 (5.75–11) (0.057)
Karyorrhexis/fibrinoid necrosis	2 (0–2)/0 (0–2) (0.089)	0 (0–2)/0 (0–2) (0.873)
Endocapillary hypercellularity	1.5 (1–2)/2 (2–3) (0.062)	3 (1.75–3)/2 (1–3) (0.573)
Cellular crescents	2 (2–4)/2 (1–4) (0.947)	4 (2–6)/2 (0.75–4) (0.329)
Subendothelial hyaline deposits	0 (0–1)/1 (0–1) (0.440)	1 (0.75–1)/0 (0–1) (0.102)
Leukocyte infiltration	0 (0–1)/1 (0–1) (0.384)	1.5 (0.75–2.25)/1 (0–1) (0.314)
Interstitial inflammation	1.5 (1–2)/2 (1–2) (0.376)	1 (0.75–2)/2 (1–2) (0.669)

Information Classification: General

CI		
CI score	1.5 (0–3.75)/2 (0–3) (0.704)	0 (0–2.5)/2 (0–3) (0.774)
Glomerular sclerosis	0 (0–1)/0 (0–1) (0.479)	0 (0–0)/0 (0–1) (0.279)
Tubular atrophy	1 (0–1)/1 (0–1) (0.396)	0 (0–1.25)/1 (0–1) (0.405)
Fibrous crescents	0 (0–0)/0 (0–0) (0.903)	0 (0–0)/0 (0–0) (0.757)
Interstitial fibrosis	0.5 (0–1)/1 (0–1) (0.807)	0 (0–1.25)/1 (0–1) (0.712)

Information Classification: General

Supplementary table 2: Comparisons of clinical features of lupus nephritis patients with and without interstitial inflammation

	Patients with interstitial inflammation (n=81)	Patients without interstitial inflammation (n=7)	*p* value
Arthralgia	32 (39.5%)	4 (57.1%)	0.363
Acute kidney injury	31 (38.3%)	0 (0.0%)	0.042
Anemia	73 (90.1%)	4 (57.1%)	0.011
Hematuria	71 (87.7%)	6 (85.7%)	0.882
Pyuria	51 (63.0%)	5 (71.4%)	0.655
Anti-nuclear antibody (+)	80 (98.8%)	7 (100.0%)	0.767
Anti-double-stranded DNA antibody (+)	68 (84.0%)	7 (100.0%)	0.251
Anti-Smith antibody (Sm) (+)	21 (25.9%)	2 (28.6%)	0.879
Anti-cardiolipin antibody (+)	5 (6.2%)	3 (42.9%)	0.001
Hemoglobin, g/l	95.5 ± 19.1	113.3 ± 15.4	0.019
Urine protein, g/24 hr	5.5 ± 5.2	3.2 ± 2.5	0.254
Serum creatinine, μmol/l	157.4 ± 102.9	75.8 ± 28.5	<0.001
C3, g/l	0.40 ± 0.17	0.37 ± 0.13	0.704
eGFR, ml/min/1.73m^2	59.4 ± 35.9	105.1 ± 23.9	0.001

Information Classification: General

Supplementary table 3: Correlations between anti-dsDNA IgE antibodies and renal histopathological scores of patients with AKI

	Patients with AKI (n = 31) median (25th percentile, 75th percentile)	Correlation between AI/CI and anti-dsDNA IgE antibodies (*r* value [*p* value])
AI		
AI score	11 (8–13)	0.349 (0.670)
Karyorrhexis/fibrinoid necrosis	0 (0–2)	0.008 (0.725)
Endocapillary hypercellularity	3 (2–3)	0.103 (0.149)
Cellular crescents	2 (2–6)	0.159 (0.302)
Subendothelial hyaline deposits	1 (0–1)	0.143 (0.133)
Leukocyte infiltration	1 (0–2)	0.181 (0.150)
Interstitial inflammation	2 (1–2)	0.095 (0.263)
CI		
CI score	1 (0–3)	0.257 (0.150)
Glomerular sclerosis	0 (0–0)	0.126 (0.253)
Tubular atrophy	0 (0–1)	0.156 (0.152)
Fibrous crescents	0 (0–0)	0.088 (0.488)
Interstitial fibrosis	1 (0–1)	0.247 (0.060)

Information Classification: General
